# ﻿Description of two new species of the genus *Spinirta* Jin & Zhang, 2020 (Araneae, Corinnidae) from southern China

**DOI:** 10.3897/zookeys.1136.94374

**Published:** 2022-12-16

**Authors:** Ke-Ke Liu, Zi-Min Jiang, Ning Ma, Wen-Hui Li, Yong-Hong Xiao

**Affiliations:** 1 College of Life Science, Jinggangshan University, Ji’an 343009, Jiangxi, China Jinggangshan University Ji'an China

**Keywords:** Corinnid, distribution, Jiangxi Province, taxonomy

## Abstract

Two new species of *Spinirta* Jin & Zhang, 2020 (Araneae: Corinnidae) from Jiangxi Province, China are described here: *S.sanxiandian***sp. nov.** (♂♀) and *S.sishuishan***sp. nov.** (♂). Detailed descriptions and photographs of the new species are provided.

## ﻿Introduction

The total number of species in the family Corinnidae Karsch, 1880 has increased greatly in the past ten years (Haddad 2013; Raven 2015; Candiani and Bonaldo 2017; Jin and Zhang 2020; WSC 2022). Now, it comprises about 824 species from 73 genera. Only nine genera with 33 species were recorded from China (WSC 2022). More than half of them are known from a single sex: eight of these were described from females and nine from males (WSC 2022). Only one corinnid species from the genus *Spinirta* Jin & Zhang, 2020 is known from Jiangxi Province.

*Spinirta* Jin & Zhang, 2020 was erected by Jin and Zhang (2020) based on ten species. Meanwhile, one *Allomedmassa* species, *A.qiaoliaoensis* Lu & Chen, 2019 (Lu et al. 2019) was transferred to the genus *Spinirta* by Jin and Zhang (2020). These species were collected from Sichuan, Chongqing, Guizhou, Hunan, Hubei, Zhejiang, and Anhui provinces and have a wide distribution in southern China. Recently, the first record of a species of *Spinirta* was reported from Wuyi Mountain in Jiangxi Province (Fan et al. 2022). However, there are still many unknown species because of the lack of systematic research and attention, especially in the southern provinces of China.

*Spinirta* spiders inhabit a wide range of habitats varying from tree canopies to the ground or leaf litter layers of the southern provinces of China (Jin and Zhang 2020; author’s pers. obs.). When we studied corinnid spiders from Jiangxi Province, we found two new species belonging to *Spinirta* and describe them herein. Furthermore, the records of corinnid species in Jiangxi Province have been updated in this work.

## ﻿Material and methods

Specimens were examined using a Jiangnan SZ 6100 stereomicroscope with a Zoom Microscope System. Both male palps and female copulatory organs were detached and examined in 80% ethanol, using an Olympus CX43 compound microscope with a KUY NICE CCD camera. All specimens treated in this work are deposited in the Animal Specimen Museum, Life Science of College, Jinggangshan University (ASM-JGSU). Measurements were taken with the AxioVision software (SE64 ver. 4.8.3) and are given in millimetres. Terminology of the male and female copulatory organs follows Jin and Zhang (2020).

Leg measurements are given as total length (femur, patella, tibia, metatarsus, tarsus). The abbreviations used in the text and figures are:

**ALE** anterior lateral eye;

**AME** anterior median eye;

**At** atrium;

**CD** copulatory duct;

**CO** copulatory opening;

**CS** cone-shaped spines;

**d** dorsal;

**E** embolus;

**EA** embolic apophysis;

**FD** fertilization duct;

**GA** glandular appendage;

**MOA** median ocular area;

**p** prolateral;

**PLE** posterior lateral eye;

**PME** posterior median eye;

**PTA** prolateral tibial apophysis;

**r** retrolateral;

**RTA** retrolateral tibial apophysis;

**Sp** spermatheca;

**St** subtegulum;

**v** ventral;

**VTA** ventral tibial apophysis.

## ﻿Taxonomy

### ﻿Family Corinnidae Karsch,1880

#### ﻿Genus Spinirta

Taxon classificationAnimaliaAraneaeCorinnidae

Jin & Zhang, 2020

921B6204-15E0-54B8-B299-06A67E4FBE76

##### Type species.

*Spinirtajinyunshanensis* Jin & Zhang, 2020. Type locality: Chongqing.

The genus includes 11 species, all of which are distributed in southern and southwest of China (WSC 2022). Currently, most of them are known only from females (three species) or males (four species) (WSC 2022). Most of China’s nine species are recorded from southwestern China (Jin and Zhang 2020). Only one *Spinirta* species, *S.wuyishanensis* Zhou, 2022 was recorded from Jiangxi Province in southeast China. It is worth mentioning that the female remains unknown.

#### ﻿ Spinirta sanxiandian

Taxon classificationAnimaliaAraneaeCorinnidae

Liu sp. nov.

**Figure 1.**
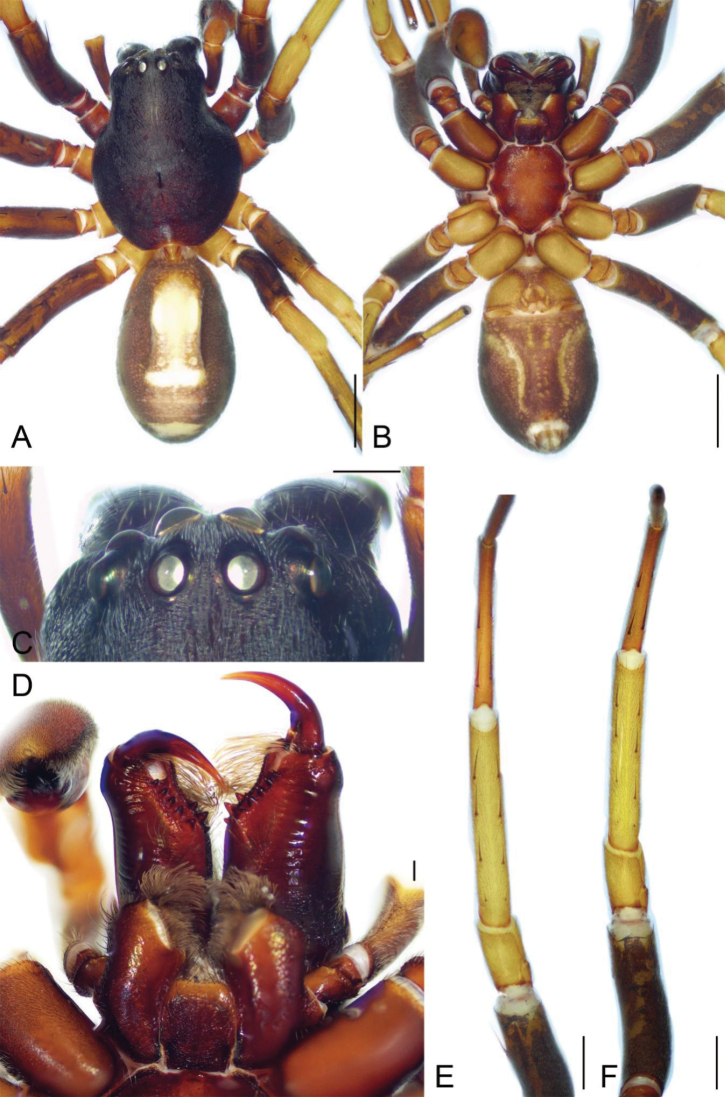
*Spinirtasanxiandian* Liu sp. nov., male holotype **A** habitus, dorsal view **B** same, ventral view **C** eyes, dorsal view **D** chelicerae, endites and labium, ventral view **E** leg I, ventral view **F** leg II, ventral view. Scale bars: 2 mm (**A, B**); 0.5 mm (**C**); 0.2 mm (**D**); 1 mm (**E, F**).

**Figure 2.**
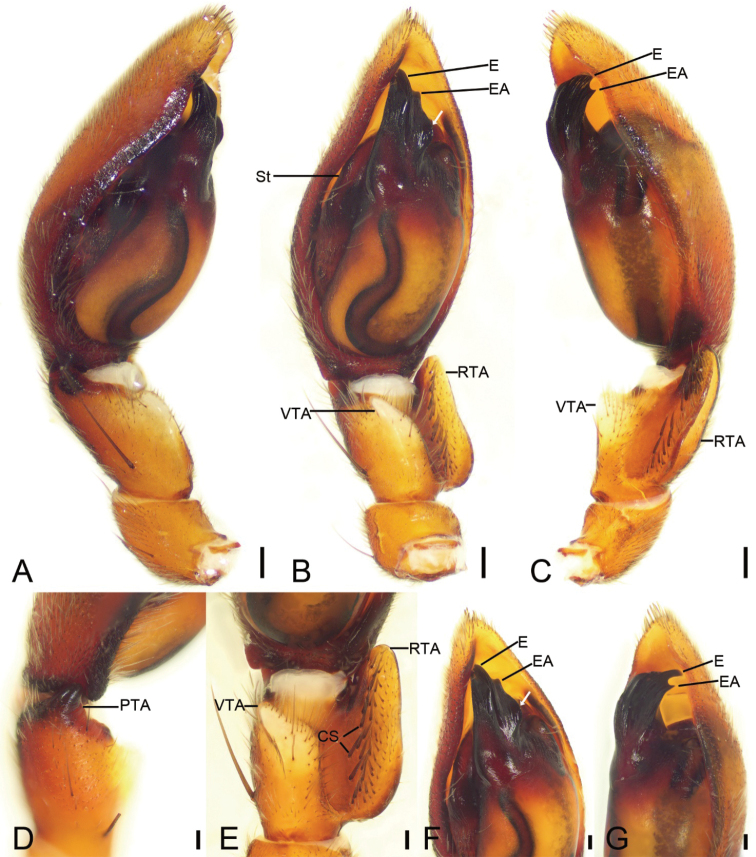
*Spinirtasanxiandian* Liu sp. nov., palp of male holotype **A** prolateral view **B** ventral view, white arrow shows the lateral apophysis located on anterolateral tegulum **C** retrolateral view **D** detail of PTA, dorso-prolateral view **E** detail of RTA, ventral view **F** detail of tegulum, white arrow shows the lateral apophysis located on anterolateral tegulum, ventral view **G** same, retrolateral view. Abbreviations: CS – cone-shaped spines, E – embolus, EA – embolic apophysis, PTA – prolateral tibial apophysis, RTA – retrolateral tibial apophysis, St – subtegulum, VTA – ventral tibial apophysis. Scale bars: 0.2 mm (**A–C**); 0.1 (**D–G**).

**Figure 3.**
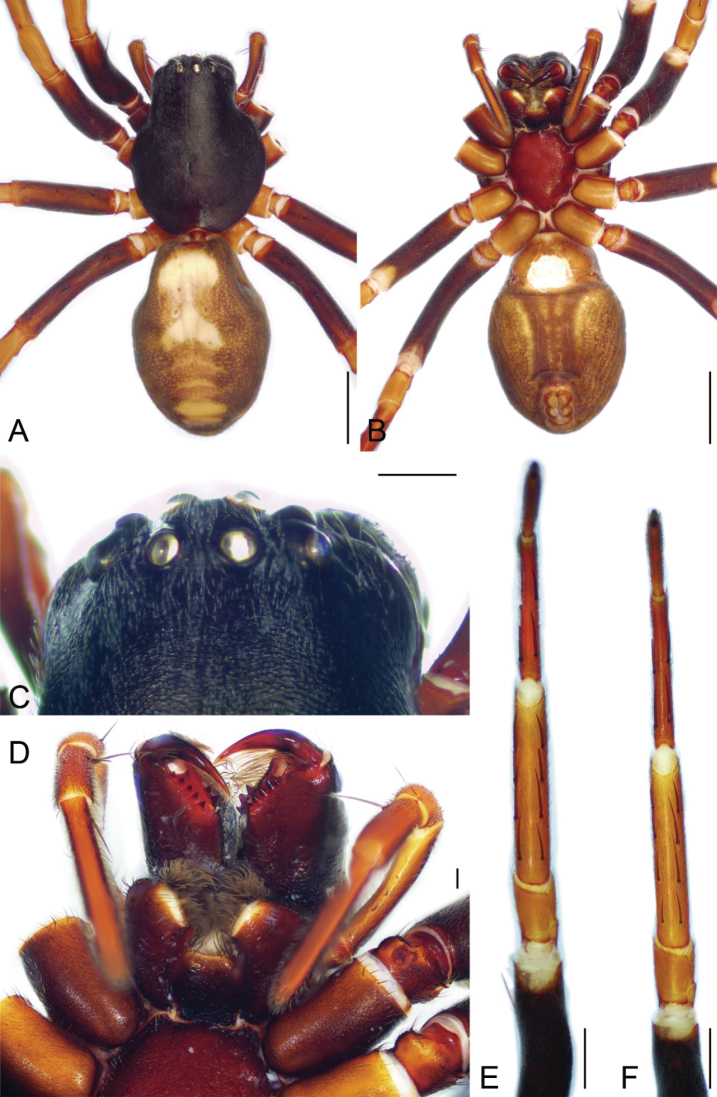
*Spinirtasanxiandian* Liu sp. nov., female paratype **A** habitus, dorsal view **B** same, ventral view **C** eyes, dorsal view **D** chelicerae, endites and labium, ventral view **E** leg I, ventral view **F** leg II, ventral view. Scale bars: 2 mm (**A, B**); 0.5 mm (**C**); 0.2 mm (**D**); 1 mm (**E, F**).

**Figure 4.**
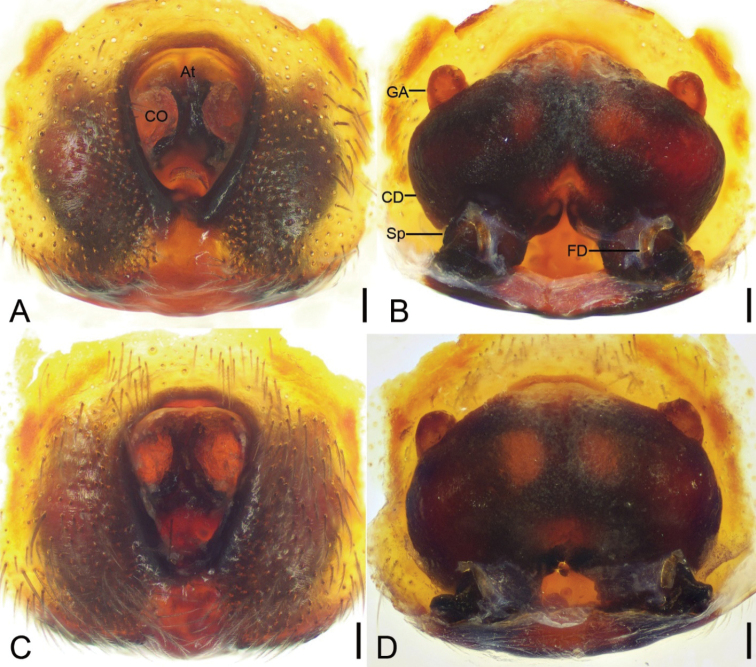
*Spinirtasanxiandian* Liu sp. nov., epigyne of female paratypes **A, C** ventral view **B, D** dorsal view. Abbreviations: At – atrium, CD – copulatory duct, CO – copulatory opening, FD – fertilization duct, GA – glandular appendage, Sp – spermatheca. Scale bars: 0.1 mm.

**Figure 5.**
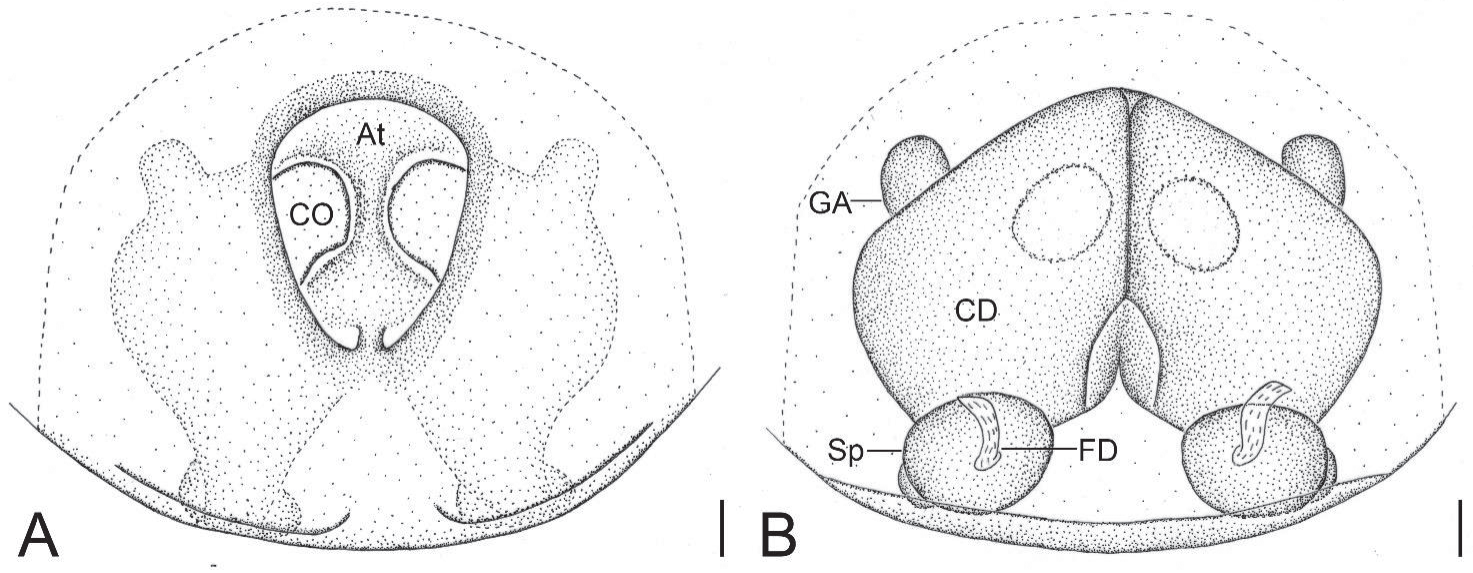
*Spinirtasanxiandian* Liu sp. nov., epigyne of female paratype **A** ventral view **B** dorsal view. Abbreviations: At – atrium, CD – copulatory duct, CO – copulatory opening, FD – fertilization duct, GA – glandular appendage, Sp – spermatheca. Scale bars: 0.1 mm.

E398719E-F60C-5419-B24C-A02AFD3BF57E

https://zoobank.org/6CEBCD02-A0B5-452B-A1AD-E0F681296427

(三仙殿刺突蛛) Figs 1 , 2 , 3 , 4 , 5 , 8

##### Material examined.

***Holotype***: 1 ♂, **China**: Jiangxi Province, Ji’an City, Qingyuan District, Donggu Town, Dawu Mountain, 26°40'48.69"N, 115°25'7.79"E, 1031 m, 25.X.2020, K. Liu et al. leg. (Cor-04). ***Paratype***: 2 ♀, 13.XI.2021, K. Liu et al. leg., other data same as holotype (Cor-03 and Cor-05).

##### Etymology.

The specific name is derived from the type locality, Sanxiandian Temple in Dawu Mountain; noun in apposition.

##### Diagnosis.

The male of this new species is similar to that of *Spinirtasparsula* Jin & Zhang, 2020 (cf. Fig. 2 vs. Jin and Zhang 2020: fig. 12B–F, 13A–D) in having the fork-like tegular apex, but can be distinguished from it by the sperm duct (*SD*) with a curved posterior part (vs. straight in *S.sparsula*) and the ear-shaped retrolateral tibial apophysis (*RTA*) without protruded base (vs. digitiform with a kidney shaped protruded base in *S.sparsula*). It also resembles *S.sishuishan* sp. nov. in having a thumb-like ventral tibial apophysis (*VTA*), a thick horn-like prolateral tibial apophysis (PTA) and a curved sperm duct (*SD*), but can be separated from it by the ear-shaped retrolateral tibial apophysis (*RTA*) (vs. shield-shaped in *S.sishuishan* sp. nov.), the anterior part of the tegulum with a broad lateral apophysis (vs. absent in *S.sishuishan* sp. nov.) and the sharp embolic apophysis in retrolateral view (vs. relatively blunt in *S.sishuishan* sp. nov.) (cf. Fig. 2 vs. Fig. 7). The female of the new species resembles *S.qizimeiensis* Jin & Zhang, 2020 in having inflated and fused copulatory ducts (*CD*) (cf. Figs 4B, D, 5B vs. Jin and Zhang 2020: fig. 22E, F). It can also be distinguished by the epigynal width/length ratio 0.88 (vs. 1.10 in *S.qizimeiensis*), the shield copulatory openings (*CO*) (vs. round in *S.qizimeiensis*), and the copulatory ducts (*CD*) extending from the anteromedial to the posterolateral part of the epigyne (vs. from anteromedial to postero-medial part of epigyne in *S.qizimeiensis*).

##### Description.

**Male.** Habitus as in Fig. 1A, B. Total length 10.87. Carapace: 5.32 long, 4.16 wide. Carapace covered with abundant short hairs. Eye sizes and interdistances (Fig. 1C): AME 0.32, ALE 0.31, PME 0.2, PLE 0.27, AME-AME 0.15, AME-ALE 0.07, PME-PME 0.3, PME-PLE 0.36, AME-PME 0.25, AME-PLE 0.47, ALE-ALE 0.87, PLE-PLE 1.45, ALE-PLE 0.2. MOA 0.74 long, front width 0.77, back width 0.72. Chelicera with three promarginal and five retromarginal teeth (Fig. 1D). Abdomen: 5.02 long, 3.31 wide. Leg measurements: I 15.48 (4.31, 1.97, 3.7, 3.52, 1.98); II 14.67 (4.25, 1.82, 3.38, 3.36, 1.86); III 12.45 (3.35, 1.62, 2.76, 3.05, 1.67); IV 16.73 (4.52, 1.83, 3.8, 4.83, 1.75); spination (Fig. 1E, F): I Fe: d2, p1; Ti: v8; Mt: v4; II Fe: d2, p1; Ti: v8; Mt: v4; III Fe: d3, p1, r1; Ti: p2, r2, v4; Mt: d2, p3, r2, v5; IV: Fe: d3, p1, r1; Ti: p2, r2, v4; Mt: d2, p3, r2, v5.

Colouration (Fig. 1). Carapace and chelicerae dark brown. Endites and labium reddish brown. Sternum red-brown. Legs: femora I–IV, tibiae and metatarsi IV dark brown to yellow, with dark brown pattern; patellae I–IV, tibiae I–III, metatarsi I–III and tarsi I–III yellow. Palps brown. Abdomen: dorsum brown, medially with a broad, longitudinal, light marking including one broad and three nearly touching transversal dark brown stripes; venter with a pair of sloping yellow stripes submedially and a pair of yellow beaded spots. Spinnerets yellow-brown.

Palp as in Fig. 2. Tibia with distinct retrolateral groove, ventral apophysis (*VTA*) thumb-like in ventral view. Retrolateral tibial apophysis (*RTA*) ear-shaped, nearly as long as tibial length, ventral surface with two lines of short cone-shaped spines (*CS*). Prolateral tibial apophysis (*PTA*) thick horn-like, strongly sclerotised, nearly as long as 1/3 of tibia. Tegulum with strongly sclerotized apex. Subtegulum (*St*) with many wrinkles on posterior surface. Sperm duct (*SD*) S-shaped in posterior part. Embolus (*E*) short, with thick base, forming a C-shape with short spine-like embolic apophysis (*EA*), nearly 3× longer than embolic apophysis.

**Female.** Habitus as in Fig. 3A, B. As in male, except as noted. Total length 10.71. Carapace: 4.84 long, 3.89 wide. Eye sizes and interdistances (Fig. 3C): AME 0.28, ALE 0.26, PME 0.19, PLE 0.24, AME-AME 0.16, AME-ALE 0.08, PME-PME 0.27, PME-PLE 0.31, AME-PME 0.26, AME-PLE 0.4, ALE-ALE 0.83, PLE-PLE 1.3, ALE-PLE 0.18. MOA 0.72 long, front width 0.66, back width 0.66. Abdomen: 5.55 long, 3.85 wide. Leg measurements: I 13.57 (3.96, 1.75, 3.19, 3.01, 1.66); II 12.5 (3.36, 1.7, 2.92, 2.92, 1.6); III 11.2 (3.09, 1.5, 2.54, 2.69, 1.38); IV 14.77 (3.92, 1.7, 3.48, 4.11, 1.56); spination (Fig. 3E, F): I Fe: d3, p1; Ti: v8; Mt: v4; II Fe: d3, p1; Ti: v8; Mt: v4; III Fe: d4, p1; Ti: p2, r2, v4; Mt: p2, r2, v5; IV: Fe: d4, r1; Ti: p2, r2, v4; Mt: p3, r2, v5.

Colouration (Fig. 3). Abdomen dark brown, medially with a broad pale mark including a broad and a thin pale chevron markings and three transversal yellow stripes.

Epigyne as in Figs 4A, B, 5. Atrium (*At*) large, shield, covers equal or less than half of epigynal plate, anteromedially located. Copulatory openings (*CO*) very large, oval, located at anterolateral atrium. Copulatory ducts (*CD*) very broad, anteriorly touching, posteriorly slightly separated. Glandular appendages (*GA*) short, located at dorsal part of copulatory ducts, extending beyond medial part of copulatory ducts, directed anteriorly. Spermathecae (*Sp*) relatively broad, separated by 1/2 width of copulatory ducts. Fertilisation ducts (*FD*) directed anteriorly, shorter than spermathecal width.

##### Variability.

The female specimens of this new species occur exactly in the same sites explored by the authors. They are identified as the same species based on appearance and epigyne. Variability was observed in the epigyne (Fig. 4), which may either have a shield/oval atrium, club-shaped or cap-like glandular appendages, and/or the anterior part of copulatory ducts closely touching or fused. The epigynal variability observed across the distribution of *Spinirtasanxiandian* sp. nov. may be the result of the influence of their development factors.

##### Distribution.

Known only from the type locality, Jiangxi Province, China (Fig. 8).

#### ﻿ Spinirta sishuishan

Taxon classificationAnimaliaAraneaeCorinnidae

Liu sp. nov.

**Figure 6.**
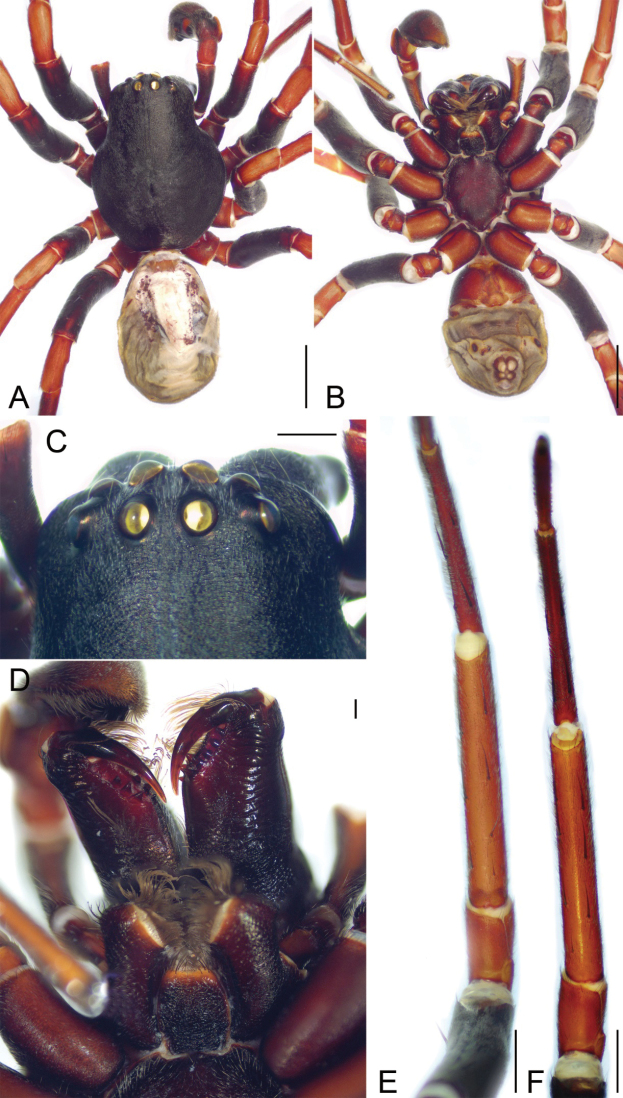
*Spinirtasishuishan* Liu sp. nov., male holotype **A** habitus, dorsal view **B** same, ventral view **C** eyes, dorsal view **D** chelicerae, endites and labium, ventral view **E** leg I, ventral view **F** leg II, ventral view. Scale bars: 2 mm (**A, B**); 0.5 mm (**C**); 0.2 mm (**D**); 1 mm (**E, F**).

**Figure 7.**
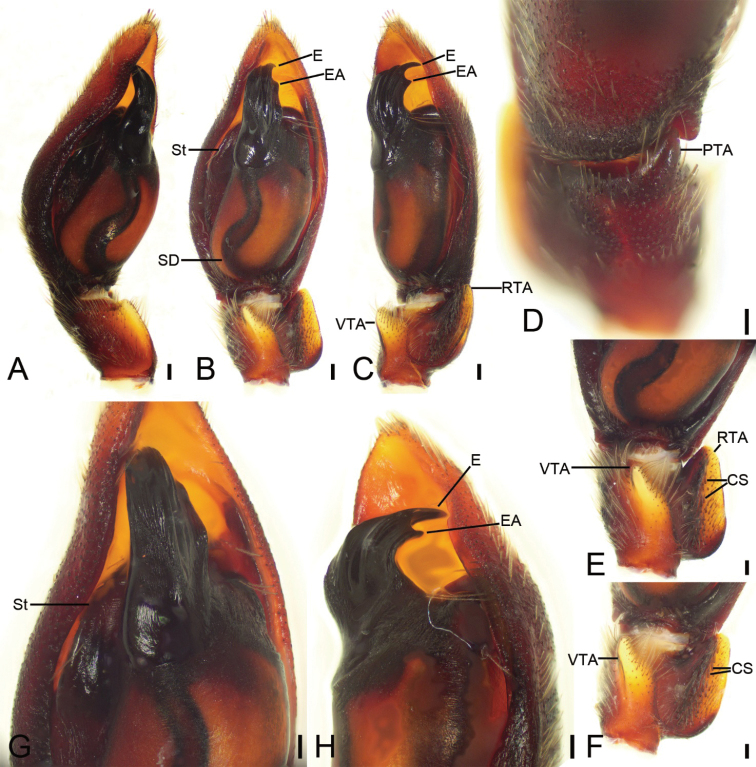
*Spinirtasishuishan* Liu sp. nov., palp of male holotype **A** prolateral view **B** ventral view **C** retro-ventral view **D** dorsal view **E** detail of VTA and RTA, ventral view **F** same, ventral view, slightly retrolateral **G** detail of tegulum, ventral view **H** same, retro-ventral view. Abbreviations: CS – cone-shaped spines, E – embolus, EA – embolic apophysis, PTA – prolateral tibial apophysis, RTA – retrolateral tibial apophysis, St – subtegulum, VTA – ventral tibial apophysis. Scale bars: 0.1 mm.

**Figure 8.**
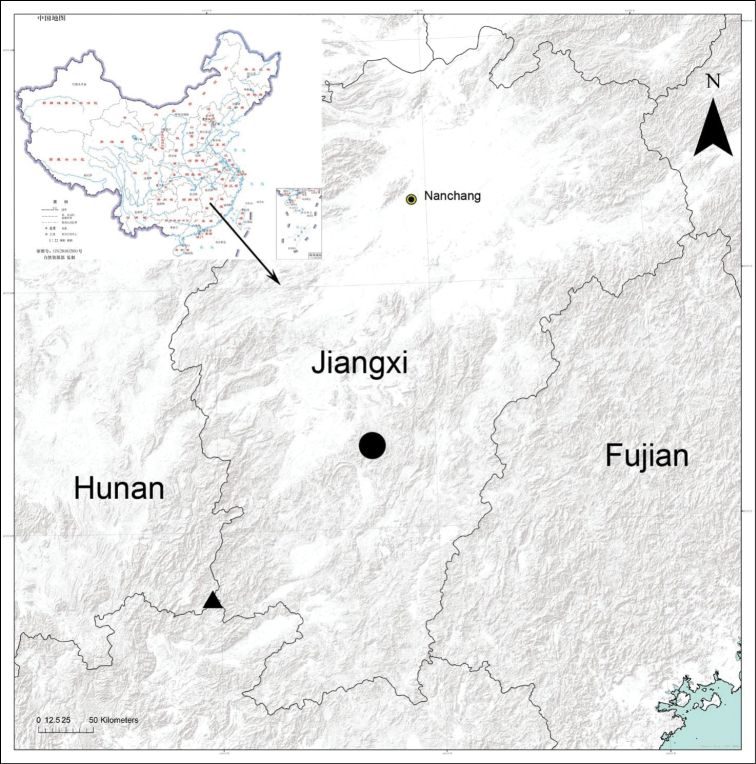
Records of *Spinirtasanxiandian* sp. nov. (circle) and *S.sishuishan* sp. nov. (triangle) from Jiangxi Province, China.

0FA58825-87CA-51AD-843C-D1560BD78095

https://zoobank.org/F06E9311-8FE0-4858-B870-22E9F59F3262

(汜水山刺突蛛) Figs 6 , 7 , 8

##### Material examined.

***Holotype***: 1 ♂, **China**: Jiangxi Province, Ganzhou City, Chongyi County, Sishui Mountain, near parking lot, 25°27'11.73"N, 113°55'30.04"E, 965 m, 2.X.2020, K. Liu et al. leg. (Cor-02).

##### Etymology.

The specific name, derived from the type locality, is a noun in apposition.

##### Diagnosis.

The male of this new species can be distinguished from *S.sanxiandian* sp. nov. by the shield retrolateral tibial apophysis (*RTA*) (vs. ear-shaped), the anterior part of the tegulum lacking lateral apophysis (vs. present in *S.sanxiandian* sp. nov.) and the relatively blunt embolic apophysis (*EA*) in retrolateral view (vs. sharp in *S.sanxiandian* sp. nov.) (cf. Fig. 7 vs. Fig. 2).

##### Description.

**Male.** Habitus as in Fig. 6A, B. Total length 10.45. Carapace: 5.6 long, 4.21 wide. Carapace covered with abundant short hairs. Eye sizes and interdistances (Fig. 6C): AME 0.35, ALE 0.24, PME 0.3, PLE 0.29, AME-AME 0.2, AME-ALE 0.08, PME-PME 0.27, PME-PLE 0.23, AME-PME 0.2, AME-PLE 0.41, ALE-ALE 0.96, PLE-PLE 1.43, ALE-PLE 0.07. MOA 0.76 long, front width 0.8, back width 0.85. Chelicera (Fig. 6D) with three promarginal and six retromarginal teeth. Abdomen: 4.79 long, 3.14 wide. Leg measurements: I 15.93 (4.62, 1.44, 4.18, 3.64, 2.05); II 15.78 (4.41, 1.9, 3.75, 3.73, 1.99); III 13.51 (3.75, 1.58, 3.15, 3.29, 1.74); IV 18.24 (4.84, 1.83, 4.29, 5.37, 1.91); spination (Fig. 6E, F): I Fe: d3, p1; Ti: v7; Mt: v4; II Fe: d1, p1; Ti: r3, v7; Mt: r2, v4; III Fe: d3, r1; Ti: p4, r2, v4; Mt: p4, r1, v5; IV: Fe: d3, r1; Ti: p3, r2, v2; Mt: p2, r2, v2.

Colouration (Fig. 6). Carapace and chelicerae dark brown. Endites and labium red-brown to dark brown. Sternum dark brown. Legs: femora I-IV dark brown, tibia and metatarus IV red to dark brown; patellae I–IV, tibiae and metatarsi I–III and tarsi I–IV red. Abdomen dark brown, medially with a pale serrulate marking. Spinnerets yellow-brown.

Palp as in Fig. 7. Tibia with distinct retrolateral groove, ventral apophysis (*VTA*) thumb-like in ventral view. Retrolateral tibial apophysis (*RTA*) shield in retrolateral view, nearly as long as tibial length, ventral surface with four lines of short cone-shaped spines (*CS*). Prolateral tibial apophysis (*PTA*) thick horn-like, strongly sclerotised, nearly as long as 1/3 of tibia. Tegulum with strongly sclerotized apex. Subtegulum (*St*) with many wrinkles on posterolateral tegulum. Sperm duct (*SD*) S-shaped in posterior part. Embolus (*E*) spine-like, with thick base, forming a C-shape with short blunt embolic apophysis (*EA*), nearly 4× longer than embolic apophysis.

**Female.** Unknown.

##### Distribution.

Known only from the type locality, Jiangxi Province, China (Fig. 8).

## Supplementary Material

XML Treatment for Spinirta

XML Treatment for Spinirta sanxiandian

XML Treatment for Spinirta sishuishan
